# Temporal validation of the CT-PIRP prognostic model for mortality and renal replacement therapy initiation in chronic kidney disease patients

**DOI:** 10.1186/s12882-019-1345-7

**Published:** 2019-05-17

**Authors:** Dino Gibertoni, Paola Rucci, Marcora Mandreoli, Mattia Corradini, Davide Martelli, Giorgia Russo, Elena Mancini, Antonio Santoro

**Affiliations:** 1 Department of Biomedical and Neuromotor Sciences, University of Bologna, Bologna, Italy; 2 Nephrology and Dialysis Unit, Ospedale S. Maria della Scaletta, Via Montericco, 4, 40026 Imola, Italy; 3 Nephrology and Dialysis Unit, Ospedale S.Maria Nuova, Reggio Emilia, Italy; 4 Nephrology and Dialysis Unit, Ospedale S.Maria delle Croci, Ravenna, Italy; 5 Nephrology and Dialysis Unit, Ospedale S.Anna, Ferrara, Italy; 6 Nephrology, Dialysis and Hypertension Unit, Policlinico S.Orsola-Malpighi, Bologna, Italy

**Keywords:** Chronic kidney disease, CKD, Prognostic models, Classification trees, Renal outcomes, Renal disease, RRT inception, Temporal validation

## Abstract

**Background:**

A classification tree model (CT-PIRP) was developed in 2013 to predict the annual renal function decline of patients with chronic kidney disease (CKD) participating in the PIRP (Progetto Insufficienza Renale Progressiva) project, which involves thirteen Nephrology Hospital Units in Emilia-Romagna (Italy). This model identified seven subgroups with specific combinations of baseline characteristics that were associated with a differential estimated glomerular filtration rate (eGFR) annual decline, but the model’s ability to predict mortality and renal replacement therapy (RRT) has not been established yet.

**Methods:**

Survival analysis was used to determine whether CT-PIRP subgroups identified in the derivation cohort (*n* = 2265) had different mortality and RRT risks. Temporal validation was performed in a matched cohort (*n* = 2051) of subsequently enrolled PIRP patients, in which discrimination and calibration were assessed using Kaplan-Meier survival curves, Cox regression and Fine & Gray competing risk modeling.

**Results:**

In both cohorts mortality risk was higher for subgroups 3 (proteinuric, low eGFR, high serum phosphate) and lower for subgroups 1 (proteinuric, high eGFR), 4 (non-proteinuric, younger, non-diabetic) and 5 (non-proteinuric, younger, diabetic). Risk of RRT was higher for subgroups 3 and 2 (proteinuric, low eGFR, low serum phosphate), while subgroups 1, 6 (non-proteinuric, old females) and 7 (non-proteinuric, old males) showed lower risk. Calibration was excellent for mortality in all subgroups while for RRT it was overall good except in subgroups 4 and 5.

**Conclusions:**

The CT-PIRP model is a temporally validated prediction tool for mortality and RRT, based on variables routinely collected, that could assist decision-making regarding the treatment of incident CKD patients. External validation in other CKD populations is needed to determine its generalizability.

## Background

The high worldwide prevalence of chronic kidney disease (CKD) [1, 2] and its burden on health care costs urges clinicians to accurately identify patients at high risk of poor prognosis. Prognostic models predicting renal failure in CKD patients have been recently developed [3–6] with the aim to facilitate effective clinical management of CKD patients, for instance timely planning of dialysis, and to achieve a more efficient cost allocation based on patients’ differential risk of renal failure and death.

In 2013 our group developed a classification tree model (hereafter named CT-PIRP) to stratify patients according to their annual estimated glomerular filtration rate (eGFR) decline. This model identified seven subgroups characterized by specific combinations of six variables (gender, age, proteinuria, baseline eGFR, phosphate levels, diabetes) which were associated with different levels of eGFR decline [7].

Because eGFR decline is correlated with kidney failure and death [8–11], we expect that the subgroups identified by the CT-PIRP model would have different risks of end-stage renal disease and of death. In community-based clinical settings in which general practitioners (GPs) are involved and advised to refer CKD patients to specialists in an early stage of the disease, eGFR decline is the main driver of adverse renal outcomes [12], because it reflects the underlying nephropathy, and patients’ adherence and response to specific therapies. However, the ability of the CT-PIRP model to predict renal replacement therapy (RRT) initiation and mortality needs to be determined. The aim of this paper is therefore to investigate the ability of the CT-PIRP model to predict RRT initiation and mortality, and to temporally validate the model on a cohort of CKD patients drawn from the PIRP project in a subsequent time interval. A validated CT-PIRP model could be very useful for nephrologists and GPs to stratify patients into clinical phenotypes at differential risks of three outcomes (eGFR decline, RRT inception and death), thereby assisting them in planning targeted follow-up strategies and treatments.

## Methods

### Data source

The study population consists of patients participating in the PIRP project [13], a collaborative network of nephrologists and general practitioners operating in Emilia-Romagna, a region of North-Eastern Italy with 4,351,393 inhabitants (2011 census data, National Institute of Statistics). The study was exempt from approval from the Ethics Committee of Emilia-Romagna. It was conducted in conformity with the regulations for data management from the Regional Health Authority of Emilia-Romagna, and with the Italian Code of conduct and professional practice applying to processing of personal data for statistical and scientific purposes (art. 20–21, legislative decree 196/2003; https://www.garanteprivacy.it/documents/10160/0/Codice+in+materia+di+protezione+dei+dati+personali+%28Testo+coordinato%29) published in the Official Journal no. 190 of August 14, 2004, which explicitly exempts the need for approval from the Ethics Committee when using anonymous data (Preamble number 8). In Italy, anonymous administrative data-gathering is subject to the law Protection of individuals and other subjects with regard to the processing of personal data, Act no. 675 of 31.12.1996 (amended by Legislative Decree no. 123 of 09.05.1997, no. 255 of 28.07.1997, no. 135 of 08.05.1998, no. 171 of 13.05.1998, no. 389 of 6.11.1998, no. 51 of 26.02.1999, no. 135 of 11.05.1999, no. 281 of 30.07.1999, no. 282 of 30.07.1999 and no. 467 of 28.12.2001) (https://www.garanteprivacy.it/web/guest/home/docweb/-/docweb-display/docweb/28335). Patients enrolled in the PIRP project provide written consent to use their anonymized data.

The PIRP project is funded by the Emilia-Romagna Region and started in 2004 with the aim to delineate intervention strategies for delaying illness progression, to increase awareness of CKD complications and to optimize CKD patient care and is still ongoing. Patients enrolled in the project receive specialized care, tailored to their severity and comorbidities, in 13 regional nephrology units to which they were referred by primary care physicians. The PIRP database collects demographic, clinical and laboratory characteristics of patients as well as their prescribed pharmacological treatment, and as of October 2018 included more than 27,000 patients.

### The derivation cohort and the CT-PIRP model

**Fig. 1:**
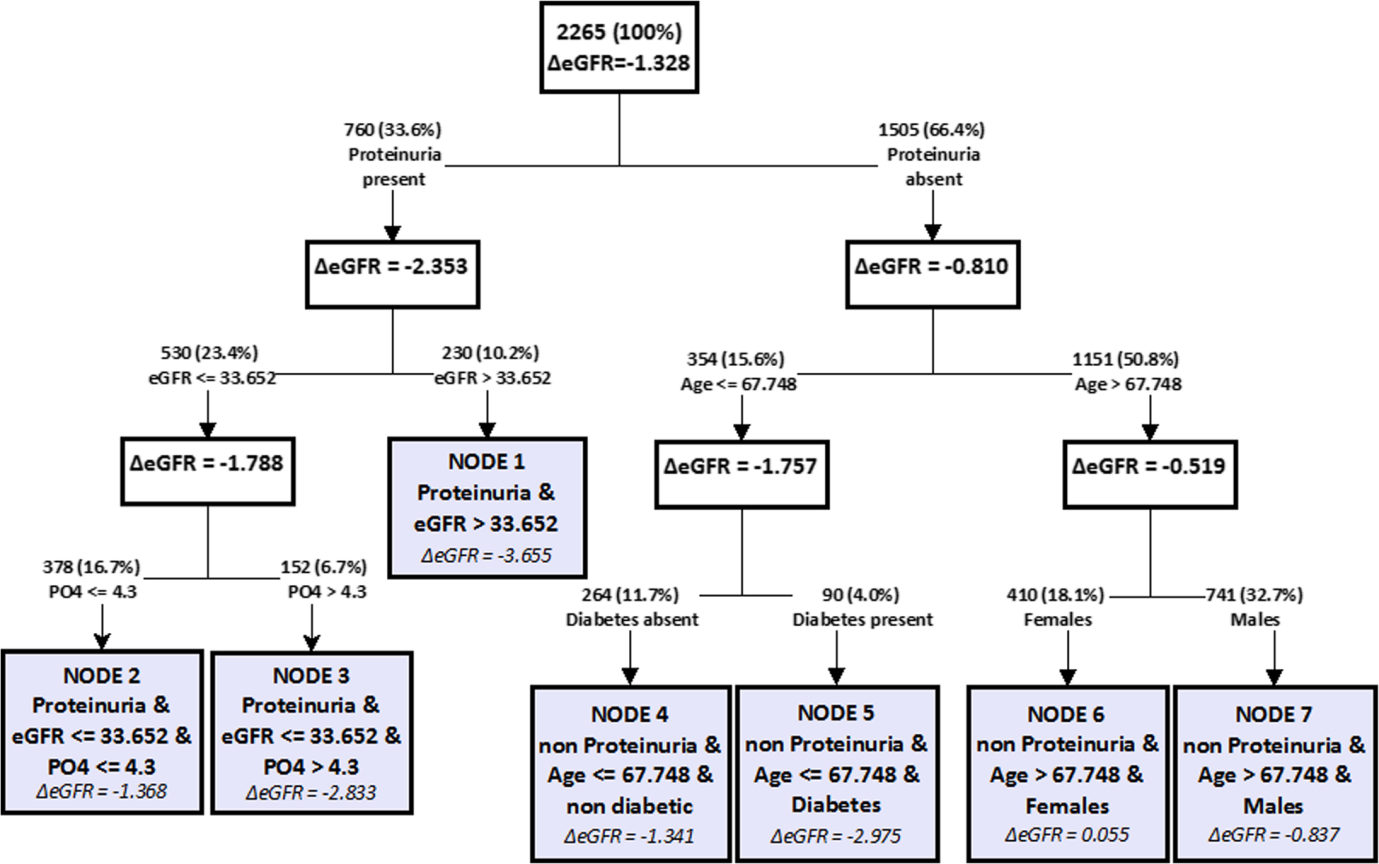
Representation of the CT-PIRP Model. Rectangles indicate subgroups of patients; in each rectangle (corresponding to a node) the mean annual estimated eGFR change is reported. The absolute and percentage frequency of each node are indicated over the arrows leading to it. Reworked figure from Rucci et al. [7]

The derivation cohort consists of 2265 CKD patients enrolled in the PIRP project from April 1, 2004 to June 30, 2010 that were followed up for at least 6 months and had at least four serum creatinine measurements between their first visit and June 30, 2011. This cohort was used to develop the CT-PIRP model, that is described in detail in a previous paper [7]. In summary, a classification tree analysis (CTA) was used to identify homogeneous subgroups of eGFR annual decline based on specific combinations of demographic and clinical characteristics. Seven subgroups of patients (also defined as nodes) with similar annual eGFR decline were identified by CTA as the result of the interactions of 6 variables (Fig. 1): proteinuria (coded as present if dipstick protein was > 20 mg/dL, or urine total protein was > 0.3 g/24 h or microalbuminuria was > 20 mg/L), eGFR, serum phosphorus, age, diabetes and gender. The other variables submitted to the CTA analysis as potential predictors of annual decline in eGFR were: hypertension, cardiac disease, smoking habits, diagnosis of nephropathy, and the baseline values of BMI, serum creatinine, serum uric acid, parathyroid hormone, glycaemia, triglycerides, LDL cholesterol, haemoglobin. The subgroup membership is a qualitative variable which embeds different patient characteristics, and as such it cannot be used to create a risk score and, moreover, the magnitude of the estimated eGFR decline alone does not fully describe the risk related to nodes.

### The validation cohort

Temporal validation assesses the performance of a prognostic model in a subsequent cohort of patients recruited from the same data source. It is the simplest form of external validation, is stronger than internal validation [14] and is widely used to evaluate the performance of prognostic models [15–17]. Thus, using the same inclusion criteria defined for the CT-PIRP model, we obtained a validation cohort from patients who entered the PIRP project between July 1, 2010 and December 31, 2016. Patients with complete data on the variables used in the CT-PIRP algorithm reported in Fig. 1 were assigned to the subgroup matching their characteristics. To enhance comparability of cohorts, we conducted a 1:1 matching of the two cohorts based on node membership and the time between the first and the last visit, rounded off to months.

### Outcomes

The outcomes of interest were inception of RRT (dialysis or transplantation, with censoring of deaths) and all-cause mortality observed until December 31, 2016. Hospital admissions subsequent to patient enrolment in the PIRP project up to April 30, 2017 were also analyzed. Information on these outcomes was obtained through linkage of the PIRP database with the hospital discharge record databases and the mortality registry of Emilia-Romagna Region.

### Statistical analysis

Patients’ characteristics of the derivation and validation cohorts were compared using the χ^2^ test or Mann-Whitney non-parametric test to take into account the non-normality of the distributions of variables. Incidence rate ratios (IRR) for RRT and mortality were used to compare the incidence of outcomes between the two cohorts.

The ability of the CT-PIRP model to predict mortality and RRT initiation was investigated in the derivation cohort using survival analysis at 6 years of follow-up. Subjects were censored on December 31, 2016 or at the date when a competing event occurred (RRT/death, loss to follow-up). Time to death or RRT inception was calculated for each subgroup using Kaplan-Meier (KM) estimate, starting at 6 months after enrolment (the minimum required follow-up time). To further evaluate the severity of illness in the subgroups of patients, the mean number of prescribed drugs (all ATC codes) and the annual number of hospital admissions after entering the PIRP project were compared across subgroups using ANOVA and Kruskal-Wallis tests, followed by post-hoc comparisons. We assigned each node a qualitative ranking based on the comparison of RRT and death risks estimated by Cox regression analyses. Very low risk was assigned when HR was less than 0.5, low risk for 0.5 < HR < 0.8, high risk when 0.8 < HR < 1.5 and very high risk when HR > 2.

The CT-PIRP model was validated in terms of discrimination and calibration. Discrimination refers to the model’s ability to identify substantially different risk profiles, while calibration indicates the predictive accuracy of the risk estimates obtained from the model [14]. As the CT-PIRP does not provide a risk score, we applied validation criteria specific for risk groups. Specifically, to evaluate discrimination we estimated RRT and mortality Kaplan-Meier survival curves of the CT-PIRP subgroups and verified whether these curves were well separated, which indicates good discrimination [18]. Both outcomes were treated as competing, applying censoring if the other outcome occurred. To evaluate calibration, we graphically compared the observed and the expected Kaplan-Meier survival curves of CT-PIRP subgroups, which should be overlapping if the model is well calibrated. The expected Kaplan-Meier curves were estimated based on the assumption that the baseline survival functions of the derivation and validation cohorts should be similar. Thus, we first estimated the baseline survival function in the derivation cohort using a Cox model with subgroup indicators as predictors; then we determined the population-average prediction in the validation cohort, by assigning to each node the corresponding baseline survival function estimated in the derivation cohort [19]. In addition, we fitted cause-specific Cox proportional hazards models for RRT and mortality in which subgroup membership, the cohort indicator and their interaction were included as predictors [20]. We expected to find some significant main effect of nodes (thus identifying high- or low-risk subgroups), possibly a significant main effect of cohort (highlighting heterogeneity in the baseline risk), but no significant interaction terms, indicating that subgroups were well discriminated regardless of the cohort of origin. The node with the largest number of outcome events was used as reference group. Robust standard errors of hazard ratios were obtained using the sandwich estimator to take into account patients’ clustering into nephrology units. To balance the length of follow-up between the two cohorts and to reduce the possible influence of long-term survivors [21], both cohorts were censored at 4 years of follow-up. The goodness of fit of these models was compared with that of other univariate Cox regression models using the baseline CKD-EPI stage or the category of annual eGFR progression rate as predictors. Lastly, we estimated the competing risks of death and RRT. This was done estimating the sub-hazard functions for RRT, mortality and loss to follow-up using the Fine and Gray model [22], and comparing the corresponding cumulative incidence function (CIF) for each node of both cohorts using stacked cumulative incidence plots. CIF represents the absolute risk for the event of interest in the presence of competing risk. Furthermore it is considered the appropriate method to take into account competing risks in prognostic models [23].

The validation process was reported according to the TRIPOD statement checklist [14]. Stata v.15.1 was used for all analyses; specifically, the user-written procedure stcoxgrp [19] was used to calculate Kaplan-Meier survival estimates.

## Results

### Predictive ability of the CT-PIRP model in the derivation cohort

**Fig. 2:**
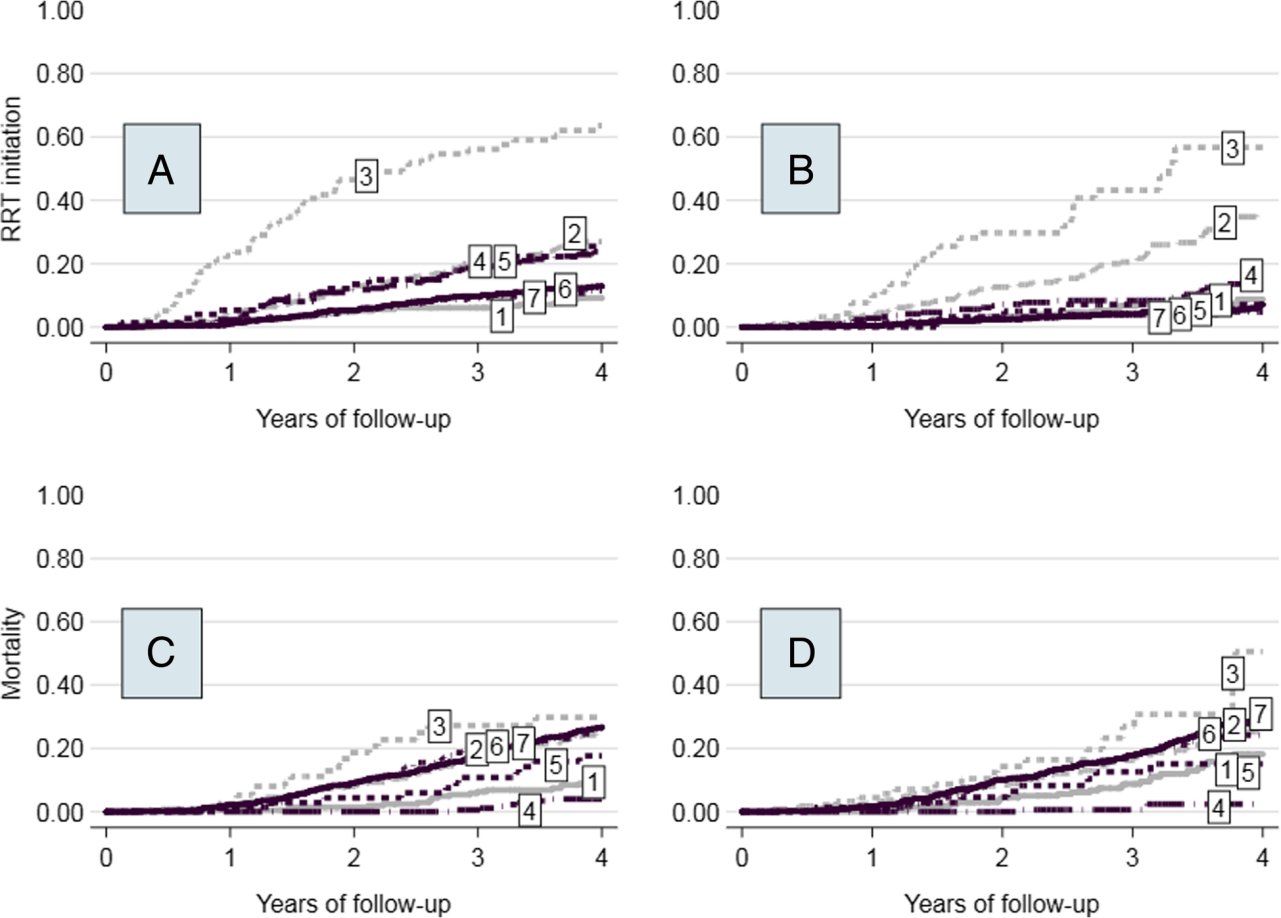
Kaplan-Meier curves of the 4-year risk of RRT initiation and mortality for the nodes of the CT-PIRP model. Panel **a**: RRT in the derivation cohort. Panel **b**: RRT in the validation cohort. Panel **c**: mortality in the derivation cohort. Panel **d**: mortality in the validation cohort. The nodes are identified by the numbers placed upon the curves

The overall mean annual eGFR decline was − 1.33 ± 5.16 mL/min (Table 1); it was faster in nodes 1, 5 and 3 (− 3.66; − 2.97; − 2.83 mL/min respectively) and slower in nodes 6 and 7 (0.06 and − 0.84 mL/min). The Kaplan-Meier failure curves (Fig. 2a) show that Node 3 had the highest risk of RRT at 6 years (71.9%), while nodes 1, 6 e 7 had similar low risks (around 19%) and nodes 2, 4 and 5 risks ranged from 32.2 to 39.0%. Cox regression hazard ratios (HR) of 2.93 (*p* < 0.001), 0.43 (p < 0.001), 0.43 (p < 0.001) and 0.45 (*p* = 0.005) were found for nodes 3, 1, 6 and 7 compared to node 2 (proteinuria patients, with eGFR ≤33.652 and serum phosphates ≤4.3 mg/dl). Mortality risk ranged between 41.1 and 49.1% for nodes 3, 6 and 7, was 35.7% for node 2, 30.0% for node 5 (Fig. 2c) and was lower for nodes 4 and 1 (9.1 and 18.0% respectively) The latter four nodes showed a significantly lower mortality risk than node 7 (non-proteinuria, older, male patients) in Cox regression. Event-free (death or RRT) median survival time varied widely from the shortest (node 3: 2.05 years) to the longest (nodes 1 and 4: 6.00 years). Patients of node 1 showed low mortality and RRT risks despite having the fastest eGFR decline; the higher baseline eGFR (46.7 mL/min) and the younger age (63.8 years) of this group might account for these results. Moreover, this group was characterized by a higher proportion of patients with diabetic nephropathies (20.9%) and glomerulonephritis (24.4%).

**Table 1:** Characteristics of the derivation cohort

	Nodes							
	All nodes	1	2	3	4	5	6	7
N (%)	2265 (100.0)	230 (10.1)	378 (16.1)	152 (4.4)	264 (10.9)	90 (3.9)	410 (19.3)	741 (35.2)
eGFR change, *mean ± sd*	−1.33 ± 5.16	−3.66 ± 6.44	− 1.37 ± 4.29	− 2.83 ± 4.05	− 1.34 ± 3.89	− 2.97 ± 6.31	.06 ± 7.02	−.84 ± 3.85
baseline eGFR, *mean ± sd*	29.0 ± 13.1	46.7 ± 13.3	23.2 ± 6.3	18.9 ± 6.8	34.0 ± 16.6	31.9 ± 14.4	24.5 ± 9.0	28.9 ± 10.9
PO_4_, *mean ± sd*	3.83 ± .83	3.60 ± .72	3.62 ± .47	5.10 ± .71	3.80 ± .88	3.99 ± .65	4.03 ± .82	3.59 ± .71
Age, *mean ± sd*	71.2 ± 12.9	63.8 ± 14.4	70.3 ± 13.0	65.7 ± 13.9	54.9 ± 11.7	61.1 ± 6.1	78.6 ± 6.0	78.2 ± 5.8
Diabetes, *n(%)*	739 (32.6)	104 (45.2)	141 (37.3)	67 (44.0)	0 (0)	90 (100.0)	118 (28.8)	219 (29.6)
Male gender, *n(%)*	1475 (65.1)	170 (73.9)	259 (68.5)	73 (48.0)	171 (64.8)	61 (67.8)	0 (0)	741 (100.0)
Number of drugs prescribed, *mean ± sd*^*a*^	8.02 ± 3.26	8.18 ± 3.0	8.84 ± 2.9	8.91 ± 2.6	6.44 ± 3.1	8.44 ± 3.9	8.24 ± 3.4	7.76 ± 3.3
Annual number of hospital admissions, *median; IQR*^*b*^	0.69; 0.78	0.67; 0.86	0.81; 0.89	1.09; 1.06	0.49; 0.80	0.94; 1.02	0.60; 0.67	0.67; 0.69
RRT events, *n(%)*	536 (23.8)	40 (17.4)	122 (32.3)	91 (60.7)	82 (31.2)	27 (30.0)	60 (14.6)	115 (15.6)
Deaths, *n(%)*	657 (29.0)	36 (15.7)	105 (27.8)	36 (24.0)	18 (6.8)	21 (23.3)	147 (35.9)	294 (39.8)
Event-free median survival time (years)	5.20	6.00	4.46	2.05	6.00	5.22	5.13	5.18
Node ranking (RRT/death)		−−/−−	+/+	++/+	+/−−	+/−	−−/+	−−/+

Abbreviations: sd = standard deviation, IQR = interquartile range

^a^ ANOVA post-hoc comparisons: node 4 < all other nodes; node 7 < nodes 2, 3

^b^ Conover-Iman test post-hoc comparison with Holm adjustment: node 3 > nodes 1, 2, 4, 6, 7; nodes 2, 5 > nodes 1, 4, 6, 7; nodes 1, 7 > 4

+ high risk, ++very high risk, − low risk, −- very low risk

### Matching and comparison of cohorts

The validation cohort comprised 3837 eligible patients, of which 2051 were matched with the derivation cohort. Matching was successful for each node in the two cohorts (Table 2) but showed some significant differences. Patients of the validation cohort had a 2.5 mL/min higher median baseline eGFR and a higher percentage with diabetes (38.1% vs 32.6%). eGFR change showed a significant but modest difference between the two cohorts only for node 5 (− 1.11 vs − 1.79 mL/min). The validation cohort showed a significantly lower incidence for RRT: IRR = 0.655 (95% CI: 0.553–0.773), which was due to the lower IRRs in nodes 4, 5, 6 and 7. Mortality was similar between the two cohorts except for node 7, that showed a significantly lower IRR in the validation cohort: IRR = 0.876 (95% CI: 0.767–0.999).

**Table 2:** Comparison of the matched derivation and validation cohorts

		Nodes							
		All nodes	1	2	3	4	5	6	7
N (%)	D	2051 (100.0)	217 (10.6)	347 (16.9)	98 (4.8)	220 (10.7)	75 (3.7)	388 (18.9)	706 (34.4)
	V	2051 (100.0)	217 (10.6)	347 (16.9)	98 (4.8)	220 (10.7)	75 (3.7)	388 (18.9)	706 (34.4)
eGFR change	D	−1.22;4.22	−2.27;5.19	−1.49;4.11	−3.25;4.72	−1.32;4.56	−1.79;5.31	−0.58;4.31	−0.86;4.15
(median; IQR)	V	−1.11;4.41	− 3.43;5.86	−2.09;4.27	− 2.00;4.94	− 0.76;4.04	− 1.11;5.17	−0.37;4.26	− 0.61;3.73
	M-W test	0.7, *p* = .482	1.8, *p* = .080	1.8, *p* = .072	−1.2, *p* = .238	−1.8, p = .072	−2.1, *p* = .040	−0.5, *p* = .647	−0.8, *p* = .414
baseline eGFR	D	27.1;16.1	42.7;13.9	23.3;10.4	17.5;10.4	31.3;23.1	30.6;16.9	24.2;11.6	28.0;15.5
(median; IQR)	V	29.6;18.7	45.5;15.1	23.1;9.6	17.5;10.4	39.3;24.0	38.2;21.7	25.4;14.1	31.8;16.6
	M-W test	−6.0, p < .001	−2.2, *p* = .030	0.7, *p* = .490	−0.1, *p* = .922	−4.2, p < .001	−3.1, *p* = .002	−2.3, *p* = .023	−5.4, p < .001
PO_4_	D	3.70;0.90	3.50;0.90	3.70;0.70	4.90;0.80	3.70;1.00	4.00;1.00	3.90;0.75	3.50;0.90
(median; IQR)	V	3.50;0.90	3.40;0.90	3.60;0.70	4.80;0.60	3.40;0.95	3.70;0.70	3.70;0.80	3.30;0.80
	M-W test	7.5, p < .001	1.6, *p* = .111	2.9, *p* = .004	1.2, *p* = .223	3.6, p = <.001	2.7, *p* = .008	4.6, p < .001	4.7, p < .001
Age	D	74.0;14.0	67.0;16.9	72.9;14.9	66.5;19.9	58.6;16.1	63.5;7.7	78.5;9.4	78.3;9.0
(median; IQR)	V	74.8;13.0	68.5;16.4	75.0;13.2	72.8;16.8	59.9;15.4	63.0;8.9	78.5;8.9	77.5;8.7
	M-W test	−1.7, *p* = .085	−1.5, *p* = .137	−2.3, *p* = .019	−2.0, *p* = .043	−1.3, *p* = .182	1.0, *p* = .297	−1.0, *p* = .300	1.2, p = .238
Diabetes	D	668 (32.6)	98 (45.2)	129 (37.2)	48 (48.9)	0 (0)	73 (100.0)	110 (28.3)	208 (29.5)
n(%)	V	782 (38.1)	118 (54.4)	164 (47.0)	47 (48.0)	0 (0)	73 (100.0)	144 (37.1)	235 (33.3)
	χ2 test	13.9, p = <.001	3.7, *p* = .055	6.8, *p* = .009	0.02, *p* = .886	–	–	6.8, p = .009	2.4, *p* = .121
Male gender	D	1340 (65.3)	161 (74.2)	235 (67.7)	49 (50.0)	139 (63.2)	50 (66.7)	0 (0)	694 (100.0)
n(%)	V	1372 (66.9)	170 (78.3)	246 (70.9)	47 (48.0)	147 (66.8)	56 (74.7)	0 (0)	694 (100.0)
	χ2 test	1.1, *p* = .291	1.0, *p* = .310	0.8, *p* = .365	0.1, *p* = .775	0.6, *p* = .424	1.2, *p* = .282	–	–

Abbreviations: D = derivation cohort; V = validation cohort; M-W test = Mann-Whitney test

Missing data were found only in the serum phosphate variable (19.4% in the derivation cohort, 19.8% in the validation cohort)

### Temporal validation for RRT

**Fig. 3:**
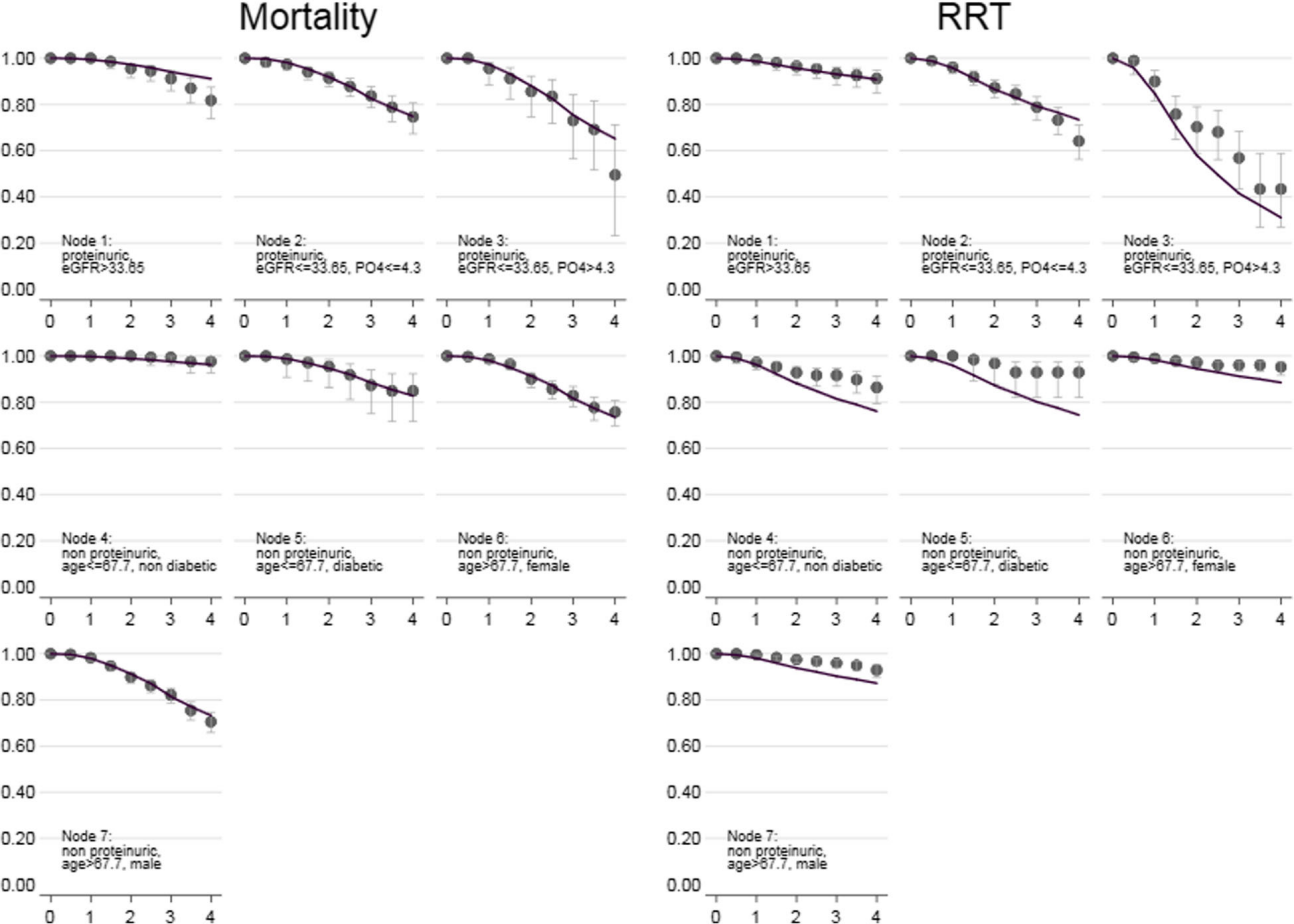
Calibration plots for the mortality and RRT initiation. For each node, lines indicate the predicted survival obtained from the Cox proportional hazard model with nodes as predictors and markers with confidence intervals indicate the observed Kaplan-Meier survival in the validation cohort

The risk of RRT initiation at 4 years estimated in the validation cohort using KM curves (Fig. 2b) proved to be similar to that of derivation cohort, and it was highest for node 3 (proteinuric patients with low eGFR and high serum phosphate) (57.8%), and low for nodes 1 (6.7%), 6 (7.0%) and 7 (5.8%). In contrast to the derivation cohort, node 2 (proteinuric patients with low eGFR and low serum phosphate) appeared as a relatively high risk group (33.7%), while nodes 4 and 5 had lower risk (12.3 and 9.2%). These findings were consistent with those obtained using the Cox regression (Table 3) in which node 3 was at higher risk (HR = 3.848, *p* < .001), nodes 1, 6 and 7 had significantly lower hazard ratios ranging from 0.308 to 0.442, and nodes 4 and 5 had similar survival than node 2, used as reference. Significant cohort X node interactions were found for nodes 4, 5, 6 and 7, indicating that in those subgroups the estimated risk was lower in the validation cohort. Calibration was not completely satisfactory, because nodes 1, 2 and 6 showed similar survival estimates (Fig. 3), while in the remaining nodes (nodes 3, 4, 5 and 7) observed and expected estimates diverged after 2 years of follow-up.

**Table 3:** Results of the Cox proportional hazards regression on time to death and time to RRT inception

	HR (95% CI)	*p*-value
Mortality		
Validation cohort	1.085 (0.886–1.328)	0.429
1	0.298 (0.237–0.376)	<0.001
2	0.932 (0.768–1.131)	0.476
3	1.379 (0.640–2.974)	0.412
4	0.122 (9.058–0.257)	<0.001
5	0.604 (0.281–1.300)	0.197
6	0.989 (0.723–1.354)	0.945
7	Ref.	
interactions		
V 1	1.766 (0.948–3.291)	0.073
V 2	0.942 (0.721–1.230)	0.658
V 3	1.129 (0.480–2.655)	0.782
V 4	0.526 (0.107–2.587)	0.429
V 5	0.878 (0.285–2.710)	0.821
V 6	0.886 (0.553–1.418)	0.614
V 7	Ref.	
RRT inception		
Validation cohort	1.196 (0.705–2.029)	0.508
1	0.308 (0.208–0.455)	<0.001
2	Ref.	
3	3.848 (2.726–5.433)	<0.001
4	0.876 (0.531–1.446)	0.605
5	0.948 (0.470–1.914)	0.882
6	0.395 (0.244–0.640)	<0.001
7	0.442 (0.262–0.744)	0.002
interactions		
V 1	0.765 (0.383–1.530)	0.449
V 2	Ref.	
V 3	0.567 (0.247–1.301)	0.181
V 4	0.441 (0.257–0.756)	0.003
V 5	0.230 (0.080–0.663)	0.007
V 6	0.379 (0.155–0.929)	0.034
V 7	0.389 (0.183–0.827)	0.014

### Temporal validation for mortality

The KM curves estimated in the validation cohort for mortality (Fig. 2d) had the same rank as those in the derivation cohort: node 4 had the lowest risk (4.2% mortality at 4 years) followed by nodes 5 (12.3%) and 1 (14.0%); nodes 2, 6 and 7 showed risks between 24.0 and 28.8%, while node 3 had the highest risk (49.5%). Cox regression was performed using node 7 as the reference (Table 3) and provided significant lower risks for node 4 (HR = 0.122, *p* < .001) and node 1 (HR = 0.298, p < .001). No significant interactions were found between nodes and cohorts, indicating that HR estimates for nodes were consistent across cohorts. Calibration was very good, because expected and predicted survival overlapped almost always perfectly (Fig. 3).

**Fig. 4:**
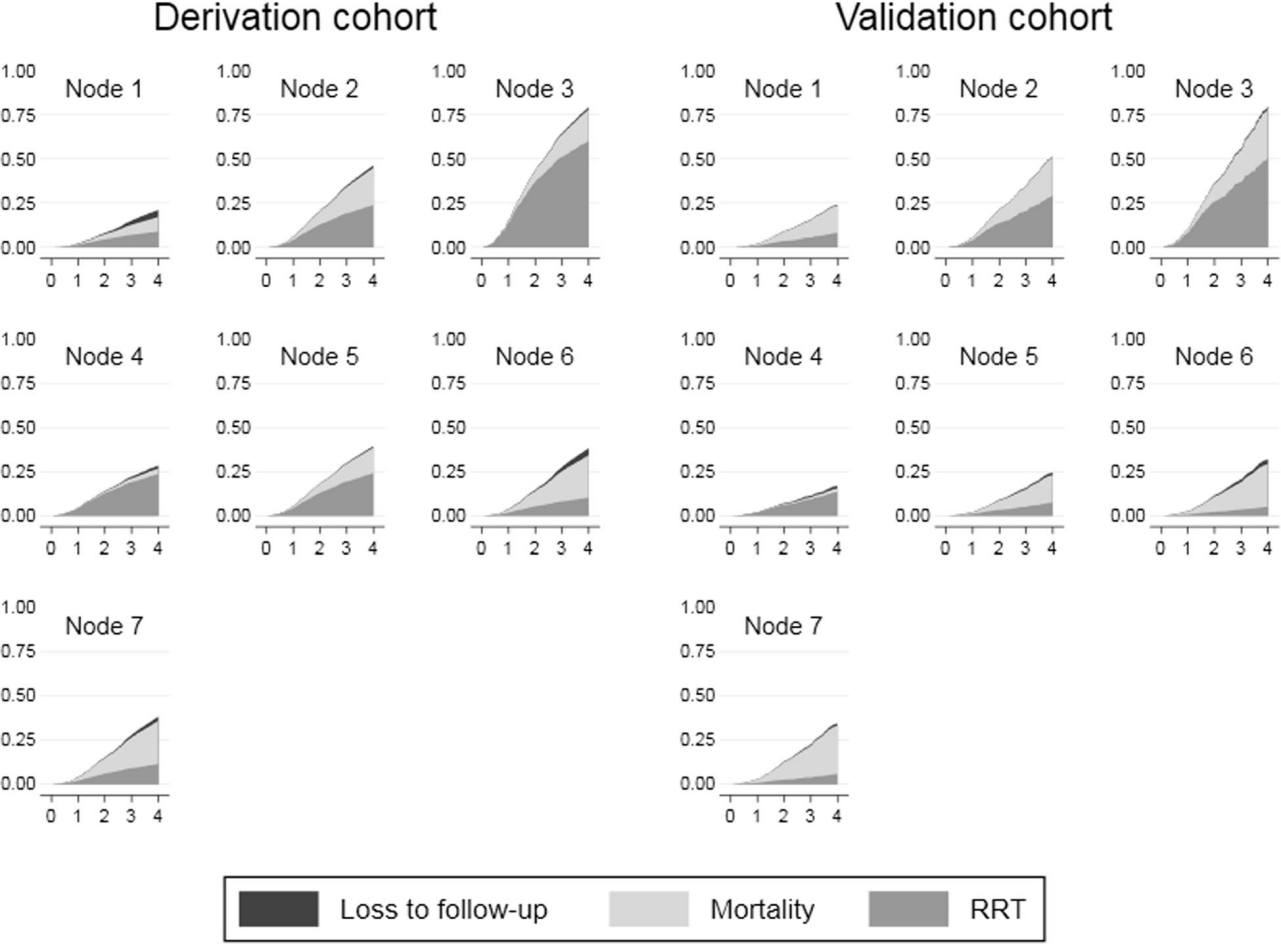
Cumulative incidence functions of RRT, mortality and loss to follow-up for each node in the matched derivation and validation cohorts

Competing risk analysis showed that the cumulative risks of adverse outcomes (CIFs) were very similar between the derivation and validation cohorts for all nodes except nodes 4 and 5, in which the estimated risk for RRT inception was lower in the validation cohort (Table 4 and Fig. 4).

**Table 4:** Results of the Fine and Gray competing risk survival analysis on time to death and time to RRT inception

	SHR (95% CI)	*p*-value
Mortality		
Derivation cohort (ref)	1	
Validation cohort	1.116 (0.911–1.365)	0.289
node		
1	0.306 (0.247–0.379)	<0.001
2	0.855 (0.715–1.023)	0.087
3	0.756 (0.380–1.507)	0.427
4	0.115 (0.054–0.245)	<0.001
5	0.565 (0.274–1.165)	0.122
6	0.982 (0.736–1.312)	0.905
7 (ref)	1	
interactions		
V 1	1.702 (0.925–3.132)	0.088
V 2	0.872 (0.667–1.139)	0.314
V 3	1.258 (0.587–2.698)	0.555
V 4	0.526 (0.109–2.533)	0.424
V 5	0.930 (0.303–2.856)	0.900
V 6	0.873 (0.568–1.341)	0.534
V 7 (ref)	1	
RRT		
	1	
Validation cohort	1.162 (0.685–1.971)	0.576
node		
1	0.334 (0.226–0.493)	<0.001
2 (ref)	1	
3	3.441 (2.537–4.666)	<0.001
4	0.975 (0.581–1-637)	0.924
5	1.002 (0.515–1.951)	0.996
6	0.391 (0.244–0.626)	<0.001
7	0.441 (0.263–0.740)	0.002
interactions		
V 1	0.754 (0.380–1.499)	0.421
V 2 (ref)	1	
V 3	0.572 (0.253–1.294)	0.180
V 4	0.442 (0.253–0.774)	0.004
V 5	0.229 (0.080–0.656)	0.006
V 6	0.383 (0.156–0.936)	0.035
V 7	0.389 (0.180–0.842)	0.017

The comparison of the goodness of fit indices of univariate Cox regression models using CT-PIRP nodes, baseline CKD-EPI stage and categories of eGFR progression rate is shown in Table 5. The CT-PIRP model fit was better than the CKD-EPI model for RRT and better than the eGFR progression rate for death.

**Table 5:** Goodness of fit comparison of univariate Cox regression models on time to death and time to RRT inception

Model	Degrees of freedom	AIC	BIC
Models on time to RRT			
CT-PIRP nodes	6	5604.325	5641.946
Baseline CKD-EPI stage	4	5638.086	5663.167
eGFR progression rate	4	5508.584	5533.665
Models on time to death			
CT-PIRP nodes	6	9427.045	9464.667
Baseline CKD-EPI stage	4	9360.737	9385.818
eGFR progression rate	4	9539.411	9564.492

## Discussion

This study provides evidence on the validity of CT-PIRP model in identifying subgroups of CKD patients with different risks of inception to RRT and death. In particular, patients with proteinuria, low baseline eGFR and high serum phosphate had the highest risk of both RRT initiation and death (node 3). On the contrary, older patients without proteinuria (nodes 6 and 7) had a relatively high risk of death and a low risk of initiating RRT. The lower mortality risk was found in non-proteinuric, younger, non-diabetic patients (node 4).

The model is extremely well calibrated for the mortality outcome, while calibration for RRT inception is poorer. In fact, the prediction of RRT for nodes 4 and 5 is not very accurate, because of the lower number of dialysis events observed in the validation cohort. Patients belonging to nodes 4 and 5 had a shorter follow-up time and a different case-mix, with higher eGFR at baseline. It is likely that with a longer follow-up the prediction accuracy of the risk of RRT initiation would improve.

Two of the six variables included in the model, eGFR and the presence of proteinuria, are widely recognized as key risk modifiers of adverse renal outcomes [8, 10, 24–28]. The use of eGFR change as a much better predictor of adverse renal outcomes than the absolute GFR value has been advocated by several authors [26, 27, 29, 30] based on the assumption that incorporates the effect of pharmaceutical-dietary treatment [31–34] and of physiological factors such as the reduced muscle mass associated with chronic illness [25, 27]. In CT-PIRP, mean eGFR change is not explicitly specified as a model parameter, however it should be seen as embedded into the definition of subgroups.

The original feature of the model is that patients are stratified through empirically-based classification criteria and not by a priori grouping, that is common practice in CKD prognostic models [10, 26, 27, 29, 30]. The CT-PIRP model does not assign individual patients a numerical risk score, but rather identifies clinical phenotypes characterized by specific interactions of six baseline variables that may guide nephrologists towards an accurate and focused examination of patients.

The CT-PIRP model is a practical tool for nephrologists, because it allows them to identify patient subgroups at greater risk of experiencing renal failure and death at 4 years from their first evaluation (Nodes 2 and 3). In these patients, treatment compliance, diet adherence and interventions on modifiable risk factors need to be enhanced and RRT can be timely planned.

Conversely, most patients at low risk of renal failure but high risk of death (nodes 6 and 7) will require greater attention in the treatment of death risk factors, in particular modifiable cardiovascular risk factors. Introducing the CT-PIRP prediction tool into clinical practice can facilitate a more personalized therapeutic approach [35].

A recent systematic review [36] pointed out that prediction models are often impractical because they require predictors rarely used in clinical practice or they lack the information necessary to perform the external validation. The CT-PIRP model does not suffer from these limitations, because the requested information is routinely collected in clinical practice and patients are assigned to the subgroups on the basis of their characteristics.

The development of different tools to identify subgroups of patients at highest risk of adverse renal outcomes that need targeted assessment and interventions has been encouraged [3, 25]. The CT-PIRP model fills the gap of the lack of predictive models for renal adverse outcomes developed in Mediterranean countries where healthcare system is mainly public and an integrated care pathway is implemented.

Our findings should be interpreted in light of some important limitations. Only patients with at least four visits and 6 months of follow-up were included in the model’s development, precluding the assessment of its prognostic accuracy in patients who rapidly reached an endpoint. The follow-up time in the validation cohort was relatively short to accurately detect the outcomes of interest in slowly progressing patients. CT methodology suffers from a limitation related to the instability of the classifier: small changes in the data may modify a tree because, if a split changes, the branches deriving from the affected node change as well. Moreover, CT is a non-parametric method that is not grounded on specific statistical assumptions and as such its decision-making procedure is algorithmic rather than statistical [37]. As a consequence, in contrast to traditional statistical modelling methods, CT do not provide scores and confidence intervals [38].

It follows that the comparison of the predictive ability of CT-PIRP with that of other traditional prognostic models based on risk scores is not straightforward [39]. The comparison of CT-PIRP model with univariate models based on stratification variables such as baseline CKD-EPI stage and classes of eGFR decline showed that the CT-PIRP nodes predict RRT better than the CKD-EPI stages and predict mortality better than the eGFR progression rate.

## Conclusions

The CT-PIRP is a promising simple prognostic model that provides an effective clinical stratification of CKD patients into subgroups at different risk of mortality and RRT, using only six variables, easily available in the current clinical practice. Thus the CT-PIRP model is applicable to most patients commonly seen in the nephrology clinics and may inform policy makers on the allocation of resources and support clinicians in the identification of patients who require a differential monitoring targeted to their risk level.

Future perspectives might include an external validation to confirm the predictive performance of the model in independent datasets.

## Abbreviations

CKD: Chronic Kidney Disease
CT: Classification Tree
CT-PIRP: Classification Tree model derived from the PIRP cohort
eGFR: estimated Glomerular Filtration Rate
HR: Hazard Ratio
IRR: Incidence Rate Ratio
KM: Kaplan-Meier
PIRP: Progetto Insufficienza Renale Progressiva (Progressive Renal Insufficiency Project)
RRT: Renal Replacement Therapy
